# Radiotherapy of primary cutaneous follicle center lymphoma: case report and review of literature

**DOI:** 10.1186/1748-717X-8-147

**Published:** 2013-06-20

**Authors:** Romana Ceovic, Ivana Jovanovic, Kresimir Kostovic, Jaka Rados, Snjezana Dotlic, Ivo Radman, Sandra Marinovic Kulisic, Davorin Loncaric

**Affiliations:** 1 Department of Dermatology and Venereology, University Hospital Center Zagreb and School of Medicine, Zagreb, Croatia; 2 Department of Dermatology and Venereology, Dr. Josip Benčević General Hospital, Slavonski Brod, Croatia; 3 Department of Pathology, University Hospital Center Zagreb and School of Medicine, Zagreb, Croatia; 4 Department of Hematology, University Hospital Center Zagreb and School of Medicine Zagreb, Zagreb, Croatia

**Keywords:** Primary Cutaneous Follicle Center Lymphoma, Superficial, Fractionated Radiotherapy

## Abstract

Primary cutaneous follicle center lymphoma is an indolent primary cutaneous B-cell lymphoma originating from the follicle center cells, composed of a combination of centrocytes (small and large cleaved cells) and centroblasts (large noncleaved cells) with a follicular, follicular/diffuse, or diffuse growth pattern. Lesions are mostly located on the head, neck and trunk. A case is presented of a 56-year-old male patient with primary cutaneous follicle center lymphoma, with lesions involving the skin of the back, shoulders, presternal area and right forearm. As the patient presented a disseminated cutaneous form of the disease that involved several anatomical regions, complete work-up was followed by superficial fractionated radiotherapy of eight fields in VI expositions, with total irradiation dose of 1400 cGy upon the following fields: right and left pectoral region, left and right shoulders, right suprascapular region, and proximal third of the right forearm. Total irradiation dose applied upon each field for the lesions located on the left and right side of the back was 1500 cGy. This therapy resulted in significant reduction of visible tumor. The patient was regularly followed up on outpatient basis for 12 months of radiotherapy, being free from local recurrence and systemic spread of the disease.

## Background

Primary cutaneous follicle center lymphoma (PCFCL) can be defined as neoplastic proliferation of the follicle germinal center cells limited to the skin. PCFCL is a primary cutaneous B-cell lymphoma composed of neoplastic B-cells with morphological and immunophenotypic properties of follicle center cells, usually a combination of centrocytes (small and large cleaved center cells) and a variable number of centroblasts (large noncleaved follicle center cells with prominent nucleoli). According to growth pattern, it is divided into follicular, diffuse and mixed pattern. This type of lymphoma is described as a separate entity in the WHO-EORTC classification of primary cutaneous lymphomas [1], as well as in the new WHO classification of hematopoietic and lymphoid tissue tumors [2]. The diagnosis of PCFCL is made in 11% of all patients with cutaneous lymphomas [1].

All cutaneous lymphomas require complete work-up including staging in order to rule out extracutaneous lymphoma of similar morphology [3]. Disease staging includes physical examination, laboratory testing, chest x-ray, ultrasonography (US) of lymph nodes and visceral organs, computed tomography (CT) of the chest, abdomen and pelvis, and bone marrow biopsy. Besides complete disease history, making an accurate diagnosis requires histopathology, immunophenotyping and molecular studies.

When extracutaneous disease has been excluded by complete work-up, the diagnosis of primary cutaneous lymphoma can be made [1,4]. Fluorodeoxyglucose-positron emission tomography (FDG-PET), recently increasingly employed, can be performed as an additional study, along with bone marrow biopsy [5,6]. As comparison of FDG-PET and bone marrow biopsy findings showed no major differences in their accuracy and specificity, the authors concluded that FDG-PET could be used as an additional study to bone marrow biopsy, in the work-up of malignant lymphoma in particular [7].

The etiology of PCFCL remains unknown, however, association with *Borrelia burgdorferi*, hepatitis C or human herpesvirus 8 infection has been occasionally described [8-11]. In the majority of cases, adults of both sexes are affected, whereas PCFCL is rare in childhood [12]. Clinical picture is characterized by the occurrence of solitary or grouped erythematous papules, plaques and tumor lesions, mostly non-ulcerated. The lesions are usually localized in the head, neck and trunk areas, less frequently on lower extremities [13-15]. The lesions localized on the back were in the past known as Crosti lymphoma or reticulohistiocytoma of the back [14]. There is no strict differentiation in clinical picture and localization of follicular and diffuse PCFCL; however, the former are predominantly found in the head and neck region, and the latter on the trunk [16,17]. Generally, PCFCL is a localized disease rarely associated with extracutaneous dissemination [18].

The histopathologic picture of PCFCL varies depending on the duration, stage of lesion growth as revealed by biopsy specimen, and localization [15,19]. Most slides show nodular to diffuse infiltrates with normal epidermis. Minor and initial lesions contain a mixed picture of centrocytes, some centroblasts, and less reactive T cells. Large centrocytes are characteristic of PCFCL. Large neoplastic B-cells may resemble fibroblasts. Follicular growth can be clearly observed in minor and/or initial lesions. Well differentiated diffuse PCFCL lesions involve the entire dermis, frequently spreading to the subcutaneous adipose tissue. The lesions are characterized by proliferation of small, medium-sized and large cleaved cells (centrocytes) admixed with a variable number of large cells with morphological characteristics of centroblasts. Small reactive T lymphocytes are mostly intertwined with tumor cells. Histologic slide with biopsy specimen of a lesion with follicular growth pattern may mimick follicular extracutaneous lymphoma [17,20]. PCFCL with follicular growth is composed of nodular infiltrates in the dermis, usually with subcutaneous tissue involvement, characterizing the follicular growth basis. If present, neoplastic follicles show morphological abnormalities such as reduced or absent mantle cell zone, reduced or completely absent stained body macrophages, and monomorphous phenomenon without clearly delineated dark and light areals. Cytomorphologically, neoplastic follicles consist of small and large centrocytes linked to centroblasts, frequently mixed with small reactive lymphocytes. In some cases, both diffuse and follicular growth characteristics are found in the same tumor; residual follicles are seen on the infiltrate periphery, while diffuse growth predominates in the central part. The morphological variants of PCFCL showing nodules of medium-sized centrocytes mixed with centroblasts without prominent interfollicular infiltrate were earlier known as large-cell lymphocytoma [21,22]. As the tumor lesion grows, neoplastic B lymphocytes also grow and replicate, while the number of reactive T cells is on a decrease [15,19]. The stromal component is usually very pronounced.

In case of either diffuse or neoplastic PCFCL, neoplastic cells are positive for CD20 and CD79a B-cell markers. In most cases of PCFCL with diffuse growth characteristics, cells are CD10- and without a network of CD21 follicular dendritic cells in the background. In contrast, in PCFCL with follicular growth cells are positive for CD10 and Bcl-6 markers [17,23-25]. The presence of small CD10+ and/or Bcl-6+ clusters outside the neoplastic follicles may be occasionally seen [17]. This phenomenon caused by ‘active migration’ of neoplastic follicular cells from the follicles toward the interfollicular space and back, has been described in nodal follicular lymphomas [26]. Other markers can be used to verify differentiation of the neoplastic cell germinal center, such as paired box gene (PAX)-5 gene and interferon regulatory factor (IRF) 8, however, other B-cells are also positive for these markers [27]. On the slides with visible diffuse and follicular growth features, CD21+ follicular dendritic cells are located in the periphery of larger areals with a diffuse growth pattern. Residual network of CD21 follicular dendritic cells is mostly found within neoplastic follicles. Unlike nodal follicular lymphoma, expression of bcl-2 is generally rare [17,27,28]. Bcl-2 positive cells are infrequently found in PCFCL, localized within the follicular center in 10%-15% of cases and only rarely in the entire neoplastic population [17,27,29-35]. Multiple myeloma oncogene-1 (MUM-1) is positive in less than 30% of PCFCL cells [36]. Polyclonal plasma cells with no restriction of κ and λ chains are frequent in patients with cutaneous lymphoma [17,37].

In most cases, PCFCL shows monoclonal distribution of the J_H_ gene, but also a reduced detection of this distribution by the PCR method. This might be, at least in part, due to the high number of somatic hypermutations characteristic of this tumor. Somatic hypermutations of variable heavy and light chains can be observed, confirming this lymphoma to originate from the follicular center cells [38,39]. Numerous literature data clearly demonstrate that PCFCL generally is not associated with t(14;18) translocation [17,27,40-44]. The presence of t(14;18) translocation is characteristic of systemic follicular lymphomas and part of systemic diffuse large cell B-lymphomas [25,28,29,40].

Inactivity of the p15 and p16 tumor suppressor genes may be detected in 10%-30% of cases [45]. In most patients with solitary or multiple lesions generally localized on the head and trunk, therapy of choice is radiotherapy based on histologic classification according to the growth pattern and number of blast cells, and the prognosis is good in these patients [1,4,13-15,46-51]. Radiotherapy is first-line therapy also for tumor lesions with histologically predominant large cleaved cells [48,50,52-55]. Skin relapses, seen in 20% of patients, which do not readily suggest disease progression, can also be treated with radiotherapy [56]. In case of localized skin lesions, excision of tumor lesions should be considered, followed by radiotherapy of the operative field and adjacent skin [15]. Chemotherapy is indicated for extensive and spread skin lesions and in patients developing extracutaneous disease [17,52]. Systemic and intralesional interferon α, or in combination with other therapeutic procedures, can also be taken in consideration [57-61].

Recent studies report on therapeutic results recorded with systemic or intralesional anti-CD20 antibody therapy in PCFCL patients [62-68]. Rituximab (anti-CD20 antibody) can be therapeutically combined with systemic chemotherapy in patients with generalized skin disease, extracutaneous disease, or relapsing cutaneous lesions [69]. Taking the growth pattern, blast cell count, and presence of solitary or multiple cutaneous lesions into consideration, PCFCL has a good prognosis with 5-year survival of about 95% [4,13-15,17,29,46-49]. There is no substantial prognosis difference between tumor lesions with a follicular growth pattern and tumor lesions with diffuse growth pattern [1,4], but some studies indicate that poorer prognosis should be expected in PCFCL cases with diffuse growth pattern, pronounced bcl-2 expression and histologically visible large cells [70].

In spite of the possible occurrence of local relapses, seen in some 20% of cases, extracutaneous dissemination of the disease is uncommon. There are only rare cases of disease dissemination to the central nervous system [70].

## Case presentation

**Figure 1:**
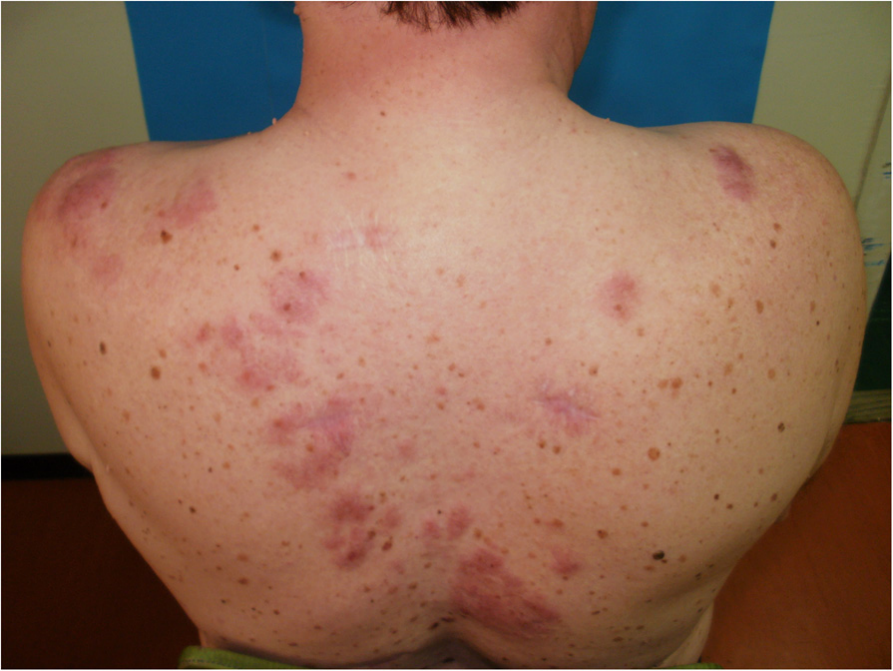
A 56-year old male patient with numerous erythematous and livid infiltrates before superficial radiotherapy.

**Figure 2:**
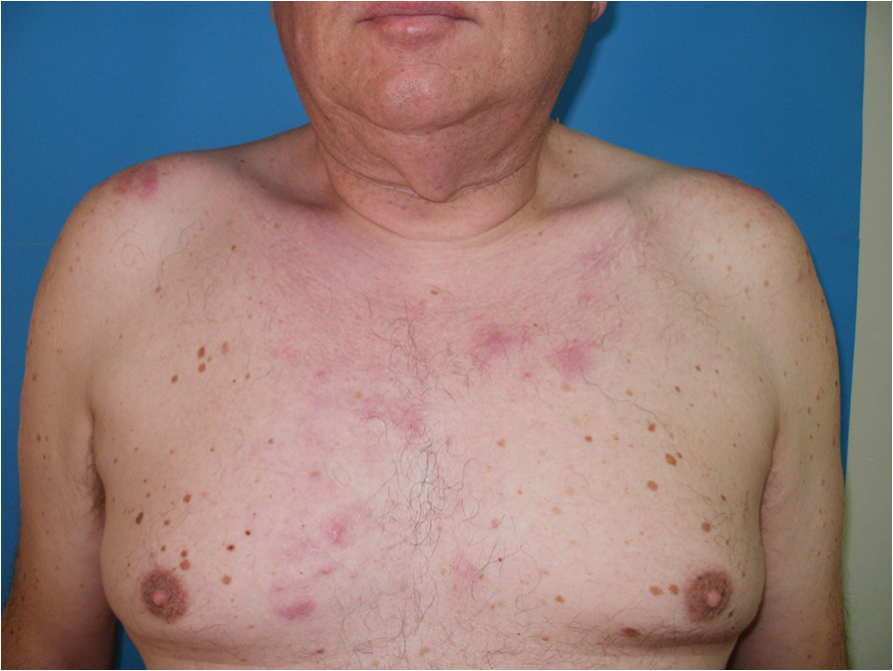
A 56-year old male patient with numerous erythematous and livid infiltrates before superficial radiotherapy.

A 56-year-old male patient presented to our Department for cutaneous lesions that had first appeared four years before. Clinical picture included numerous erythematous and erythematous-livid infiltrates, 2–8 cm in diameter, on his upper back, shoulders, in the presternal region and in the proximal third of his right forearm [Figures 1 and 2]. There was no enlargement of the palpable lymph nodes and the patient denied any subjective discomforts of pain or itch. He had been treated for type 2 diabetes mellitus and arterial hypertension for years and regulary checked for multipli naevi pigmentosi on the trunk.

Laboratory findings: complete blood count, urine, blood glucose, liver enzymes, bilirubin, urea, creatinine, lipid profile, creatine kinase, lactate dehydrogenase, C-reactive protein, protein electrophoresis, immunoglobulins, and serum Cu were within the reference values. Serology for hepatitis C and B viruses and *Borrelia burgdorferi *[5-8], and HIV-ELISA produced negative findings.

**Figure 3:**
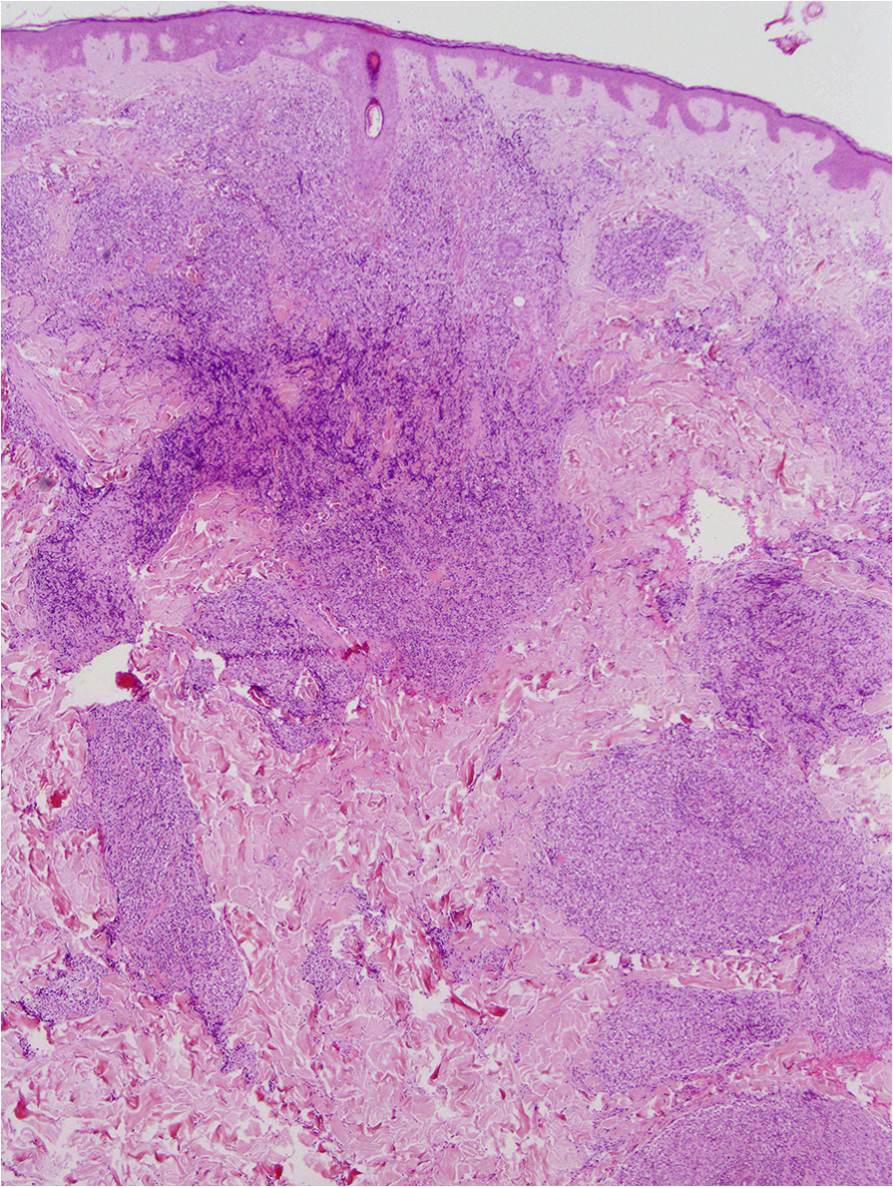
**The epidermis is preserved, with diffuse and nodular infiltrates of lymphoid cells.** (H&E, magnification 40×).

**Figure 4:**
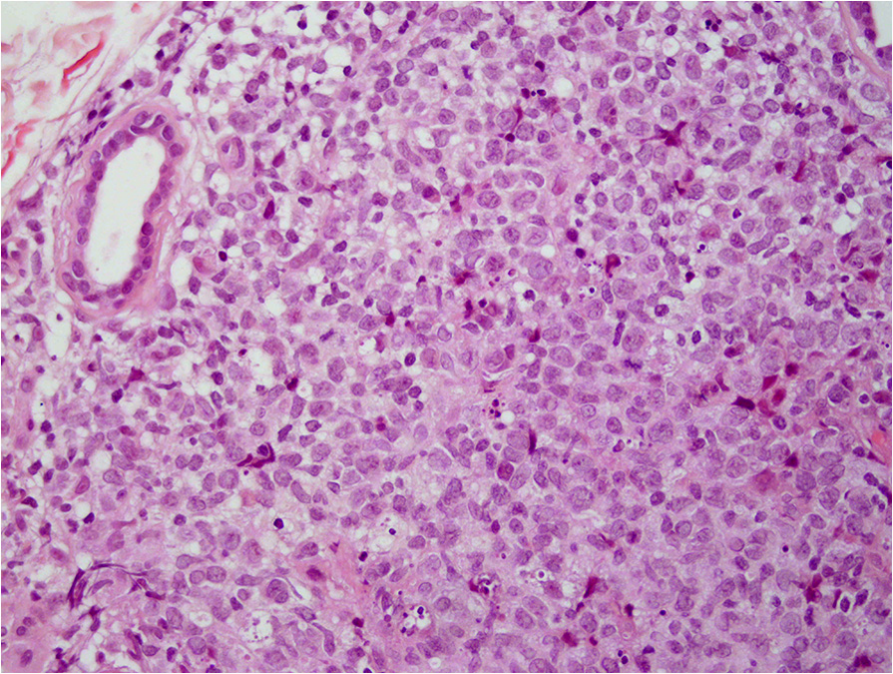
**The infiltrate is composed of atypical, medium-sized and focally large lymphatic cells.** (H&E, magnification 400×).

Biopsy specimens were obtained from lesions on the left shoulder, the back and the right shoulder. Histopathology of all three specimens revealed superficially regular, preserved epidermis and diffuse full-depth lymphocyte infiltration of the excised dermis [Figure 3]. In the deep dermis, there was abundant lymphocytic infiltrate with the formation of follicular germinal center and the surrounding ‘mantle zone’. The infiltrate was composed of atypical, medium-sized and focally large lymphatic cells [Figure 4].

**Figure 5:**
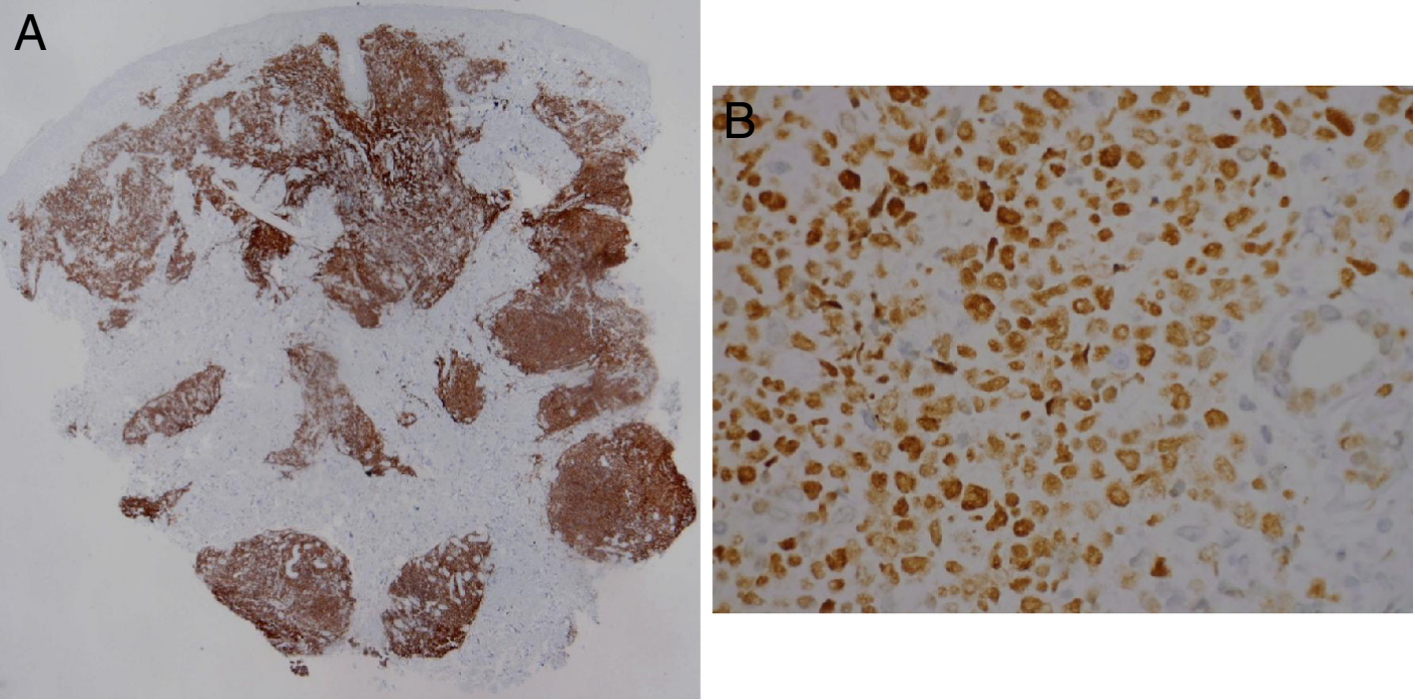
Immunohistochemical staining for CD20 demonstrates diffusely positive reaction in tumour cells (A: IHC, CD20, magnification 20×), coexpressing BCL6 (B: IHC, BCL6, magnification 400×).

Immunohistochemistry showed the atypical, medium-sized and focally large lymphatic cells to be CD20+, bcl-6+, bcl-2 weakly positive, CD10-, MUM1-, CD3- and CD5- [Figure 5A,B]. Staining for κ and λ chains demonstrated rare polyclonal plasma cells peripherally. Staining for CD21 showed the follicular dendritic network to be preserved and focally extended.

Clonal B lymphocyte population was demonstrated by molecular analysis using the PCR method with analysis of rearrangement in the gene for immunoglobulin heavy chain and the gene for immunoglobulin light chain of kappa type (IgK).

Considering the histologic picture and verified clonality, the finding corresponded to the skin infiltration with B-immunophenotype lymphoma, requiring further exclusion of extracutaneous lymphoma [1,3,4].

The patient was referred to a hematologist; based on hematologic examination and the findings available, the hematologist suggested ruling out systemic disease and recommended bone marrow biopsy and multi-slice computed tomography (MSCT).

Bone marrow biopsy revealed no evidence of tumor.

MSCT of the thorax, abdomen and pelvis showed no lymphadenopathy, however, visualizing multiple concrements up to 8 mm in size in the cholecyst.

Upon complete work-up, and considering the histologic picture, verified clonality and exclusion of extracutaneous spread of the disease, the finding was consistent with the picture of PCFCL.

**Figure 6:**
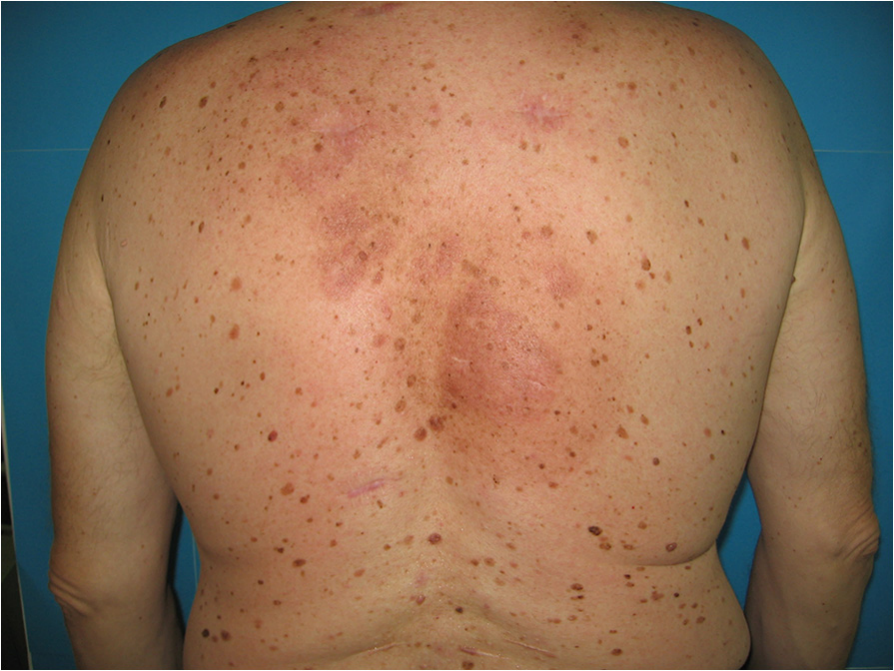
Post-radiotherapy appearance.

**Figure 7:**
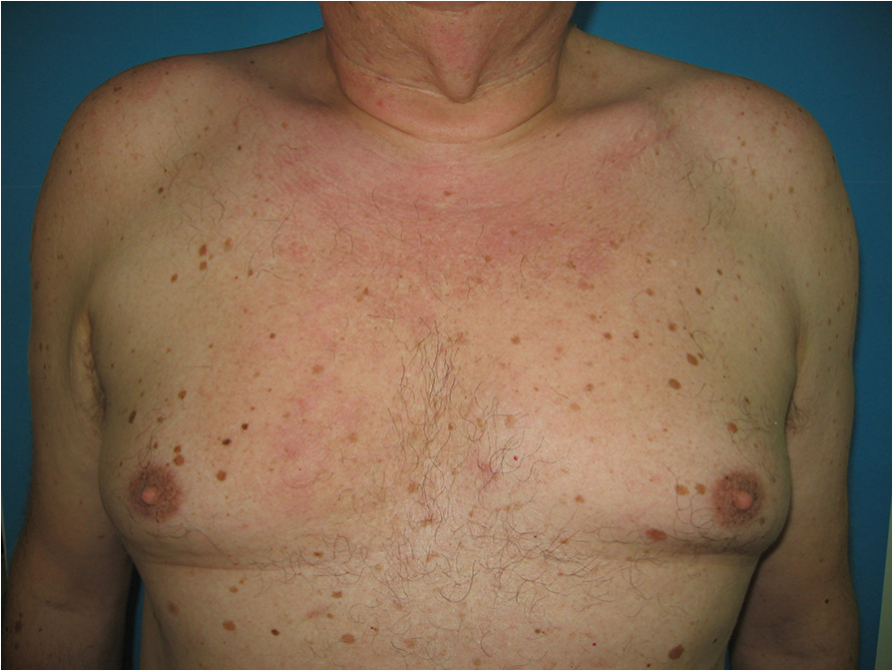
Post-radiotherapy appearance.

As our patient had a disseminated cutaneous form of the disease involving multiple anatomic regions, we decided on the use of superficial fractionated radiotherapy [1,4,13-15,46-51], administered upon eight fields in VI expositions, with total irradiation dose of 1400 cGy, on the following irradiation fields: left and right pectoral region, left and right shoulder, right suprascapular region, and proximal third of the right forearm. Total dose received *per* field upon lesions on the left and right side of the back was 1500 cGy. This therapy resulted in significant reduction of visible tumor [Figures 6 and 7. Post-radiotherapy skin appearance]. The patient was regularly followed up for *12* months following radiotherapy and remained free from local relapse or extracutaneous dissemination of the disease. Staging of the disease can be repeated in case of relapse or every 12 months (heart and lung x-ray is usually repeated every 2–3 years).

## Conclusion

In the patient presented, the diagnosis of PCFCL with follicular growth pattern was made by complete work-up and exclusion of systemic disease. Although it is an indolent B-cell lymphoma, appropriate approach to the patient and complete diagnostic work-up are necessary as in all cutaneous lymphomas. Close collaboration of specialists in dermatology, pathology, cytology and hematology is of utmost importance to reach an accurate diagnosis, to perform proper disease staging, and to choose the most suitable therapeutic modality.

Of the known therapeutic procedures, we decided to use superficial fractionated radiotherapy upon eight fields, which led to the significant reduction of visible tumor.

Patients with this type of cutaneous lymphoma have good prognosis, with the expected 5-year survival of 95% and rare systemic disease development. Skin relapses are reported in 20% of cases and are treated with radiotherapy. Our patient has been on regular follow up for possible skin relapses or extracutaneous disease dissemination.

### Consent

Written informed consent was obtained from the patient for publication of this Case report and any accompanying images. A copy of the written consent is available for review by the Editor-in-Chief of this journal.

## Competing interests

The author(s) declare that they have no competing interests.

## Authors’ contributions

RC, IJ and KK are doctors who were treating patient from the first day he came to the Clinic. They have made substantial contributions to his treatment, acquisition of data, analysis and interpretation of data. They have been involved in drafting the manuscript or revising it critically for important intellectual content; and have given final approval of the version to be published. JR,SD and DL are dermatopathologists who made histopathology and immunohistochemistry, SMK was patient’s leading doctor during hospitalization, IR is hematologist who have made substantial contributions to conception of diagnostic procedures, analysis of data and treatmant. All authors read and approved the final manuscript.

## Authors’ information

Assist. Professor Romana Ceovic is Head of the Department of Dermatological Oncology at the University Hospital, Zagreb, Croatia and Head of Refferal Center for Dermatooncology in Croatia. Her current interests include radiotherapy of skin tumors.
